# Gout involved the cervical disc and adjacent vertebral endplates misdiagnosed infectious spondylodiscitis on imaging: case report and literature review

**DOI:** 10.1186/s12891-019-2813-8

**Published:** 2019-09-14

**Authors:** Suying Zhou, Yundan Xiao, Xin Liu, Yi Zhong, Haitao Yang

**Affiliations:** Department of Radiology, the First Affiliated Hospital of Chongqing Medical University, 1 Youyi Road, Yuzhong District, Chongqing, 400016 China

**Keywords:** Spine, Cervical, Gout, Radiology

## Abstract

**Background:**

Gout in spine is rare and commonly mimics some infectious or tumoral lesions, the differentiation of spinal gout from other diseases is not always easy. We report a case of gout involved cervical disc and adjacent vertebral endplates whose etiology was initially not determined. Compared with the previous published 10 similar cases, this case displayed a complete and continuous image data with higher image quality and resolution than before. So we give a brief literature review for concerning cervical gout, with the emphasis on the discussion of radiological findings.

**Case presentation:**

A 50-year-old male with a 5-year history of neck and shoulder pain had muscle atrophy and weakness in both arms. Physical examination revealed multiple tophi were seen in left wrist, both feet and knee; bilateral superficial sensory declined below level of mastoid portion and the muscle strengths of limbs decreased. Laboratory findings showed hyperuricemia and the C-reactive protein level was very high. Imaging studies including Computed Tomography (CT) and Magnetic Resonance Imaging (MRI) showed abnormality of the C5–6 intervertebral disc and irregular osteolytic destruction of both adjacent C5–6 endplates, narrowing of C5–6 disc space and swelling of prevertebral soft tissue. Under the circumstance of the lesions being not determined and nerve root symptoms, surgical treatment was performed and pathological examination of the specimen revealed deposited uric acid crystals surrounded by granulomatous inflammation. After surgery combined with pharmaceutical and rehabilitation treatment, the muscle strengths of limbs, the pain of neck and shoulder and the level of serum uric acid were all improved.

**Conclusions:**

Cervical spinal gout involving the disc and adjacent vertebral endplates is uncommon and may misunderstand infectious spondylodiscitis. Physician and radiologist should take the gouty spondylitis into account with a combination with previous history and clinical manifestations when encountering with such this condition.

## Background

Gout is a metabolic disorder characterized by hyperuricemia and abnormal depositions of urate around a variety of tissues, more frequently including peripheral joints of the upper and lower extremities, typically in the first metatarsophalangeal joint [1]. Gouty involvement in the axial skeleton is a seemingly uncommon manifestation.

Any segment of the spine and its components (vertebral bodies, pedicles, lamina, ligaments, interapophyseal cartilage, and epidural and intradural spaces) may be involved, with lumbar involvement being the most common [2, 3]. It has been reported by Michael et al. that most cases (80.6%) only involved one region of the spine and 24.8% affected only the cervical spine [1]. Gout involving the endplates of two contiguous vertebral bodies and intervertebral disc is rare, which frequently mimics some degenerative changes and infectious spondylodiscitis on imaging, leading to delayed diagnosis and treatments. Therefore, the main imaging differentiation factors between these conditions should be further investigated. Up to now, there have only been 10 cervical gout cases with images reported in the English-language literature searched in PubMed [3–11]; most of them showed the incomplete image sequence and poor image quality which were insufficient to understand the imaging manifestations of this condition for improving diagnostic accuracy. In this article, we report a case about a spinal gout affected the cervical disc and adjacent endplates with a complete and continuous image data with higher image quality and resolution than previous published cases, whose etiology was initially not completely determined and suspected as infectious spondylodiscitis, and provide a brief literature review concerning cervical gout. In addition, the case report is prepared and reported in accordance with CARE-checklist [12].

## Case presentation

### Patient information

A 50-year-old Chinese man with a 5-year history of neck and shoulder pain presented with muscle atrophy and weakness in both arms. He was admitted to hospital with complaints of unstable holding for 9 months and numbness of limbs for 2 months, which worsened in one week. Five years previously, he tried the physiotherapy due to the pain of neck and shoulder, and relief from pain followed. Nine months previously, muscle atrophy and weakness in both arms were noted but without being promptly treated. And his pain of neck was obviously worsened 2 months before admission, together with numbness of limbs; the cervical and lumbar image examinations in other hospital were suggestive of protrusion of intervertebral disc at the C5–7 level and the L4-S1 level. Then he received the physiotherapy and felt the relief of pain, but numbness of limbs did not significantly relieve, which suddenly worsened in one week.

The patient also presented with a 3-year history of diabetes mellitus (DM), a 20-year history of gout involving left wrist and left knee, a 30-year history of smoking and drinking. Previous pharmaceutical treatment for gout had been intermittent for about 1 year, consisting of dexamethasone for acute episodes. Patient’s family and psychosocial history were not relevant for this case report.

### Clinical findings

After admission, physical examination was performed in accordance with best practice and clinical guidelines [13] by physician, in which multiple tophi were seen in left wrist, both feet and knee; bilateral superficial sensory declined below the level of mastoid portion, particularly below the knee, which presumably resulted from the lumbar lesions. The muscle strengths of upper limbs were graded 2+ to 4 and lower limbs were graded 0 in accordance with Medical Research Council (MRC) Scale for Muscle Strength [14]. The reflex of bilateral biceps, triceps and radial periosteum disappeared; the reflex of bilateral knee was normal and that of bilateral ankle disappeared. The muscle atrophy of bilateral thenar, hypothenar, forearms, biceps, triceps deltoid and quadriceps femoris, triceps surae was also noted. The gout history and physical examination revealing tophi in multiple joints informed the physician to perform some biochemical tests relating to gout, which found hyperuricemia with a level of 542 umoles/L (normal range, 208–428 umoles/L), and the C-reactive protein was 125 mg/L (normal range, 0–8 mg/L).

**Fig. 1:**
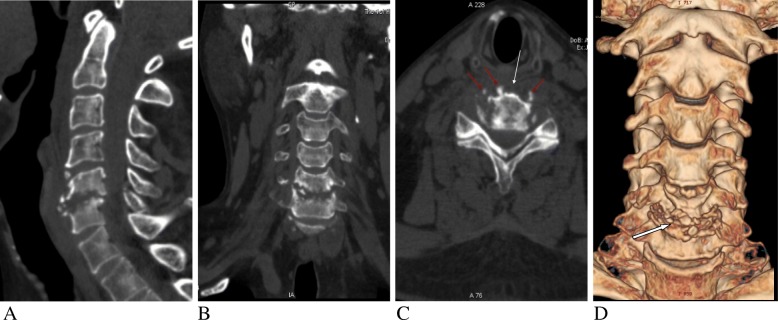
CT image of gout involved the cervical disc and adjacent vertebral endplates in a 50-year-old man with progressive muscular atrophy and weakness in both arms for 9 months and numbness of limbs for 2 months. **a** Sagittal reconstruction of the cervical spine CT shows slightly irregular osteolytic destruction of C5–6 endplates with discontinuous peripheral sclerotic margins and multiple sequestra and calcification, as well as the narrowing of C5–6 disc space. **b** Coronal reconstruction CT image illustrating the same findings. **c** Axial CT image shows irregular marginal destruction of the vertebral body (white arrow) with multiple small sequestra and calcification (red arrows). **d** Volume rendered (VR) image shows multiple sequestra (white arrow) and the narrowing of disc space

**Fig. 2:**
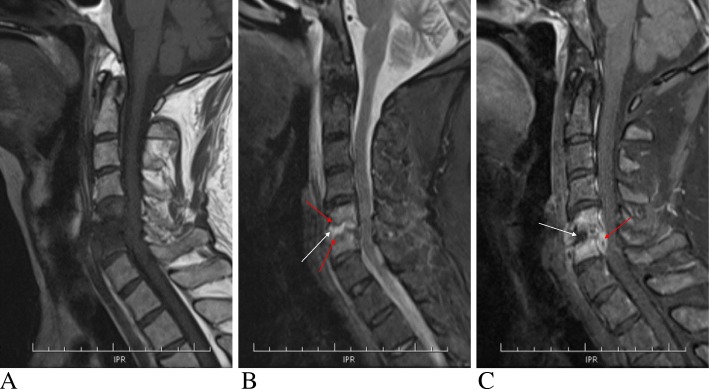
Magnetic resonance (MR) imaging findings in the patient. **a** Sagittal MR T1-weighted image of the cervical spine showing the normal bone marrow and endplates signal disappeared and replaced by diffused low-signal intensity of the C5 and C6 vertebrae. **b** Sagittal short time inversion recovery (STIR) MR image illustrating the same diffused high signal intensity of the C5–6 disc (white arrow), the waved erosion and destruction of both adjacent C5–6 endplates (red arrows), and the swelling of prevertebral soft tissue. **c** Contrast-enhanced sagittal MR T1-weighted image with fat saturation illustrating significant enhancement of C5–6 vertebrae marrow with no enhancement of the anterior disc (white arrow), and spinal dural thickening and enhancement (red arrow)

Based on the symptoms and physical examination, physician intended to consider peripheral neuropathy and could not rule out cervical spondylotic myelopathy. Then the cervical computed tomography (CT) and contrast enhanced magnetic resonance imaging (MRI) were performed. CT scan showed the slightly irregular osteolytic destruction of the C5–6 endplates with discontinuous peripheral sclerotic margins and multiple small calcification and sequestra. Narrowing of C5–6 disc space and mild spinal stenosis were also found (Fig. 1). MR images displayed diffused abnormal signal of the C5 and C6 vertebrae marrow, which were low signal intensity on T1-weighted images and intermediate to high signal intensity on T2 and short time inversion recovery (STIR) images. The erosion and destruction of both adjacent C5–6 endplates showed discontinuous and waved changes. The C5–6 intervertebral disc appeared fluid like signal changes that were hypointense on T1 and hyperintense on T2-weighted images. Swelling of prevertebral soft tissue was also found. Contrast-enhanced images showed significant enhancement of the C5–6 vertebrae marrow with no enhancing intervertebral disc. Spinal dural thickening and enhancement of the C5–6 level could be also seen on the enhanced T1-weighted images (Fig. 2).

### Timeline

**Fig. 3:**
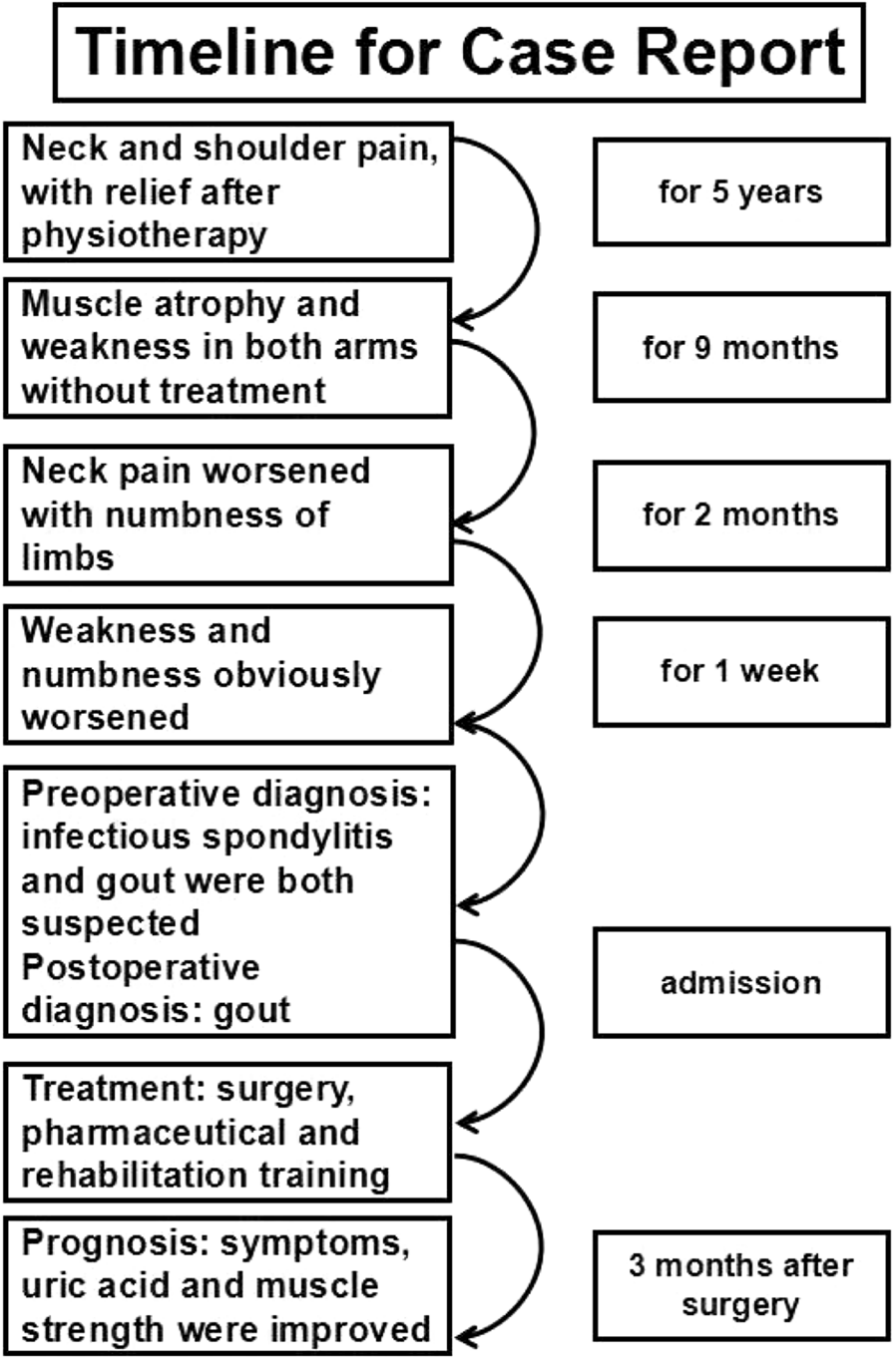
Timeline of case report. The timeline for the development of this case is summarized in Fig. 3

The timeline for the development of this case is summarized in Fig. 3.

### Diagnostic assessment

**Fig. 4:**
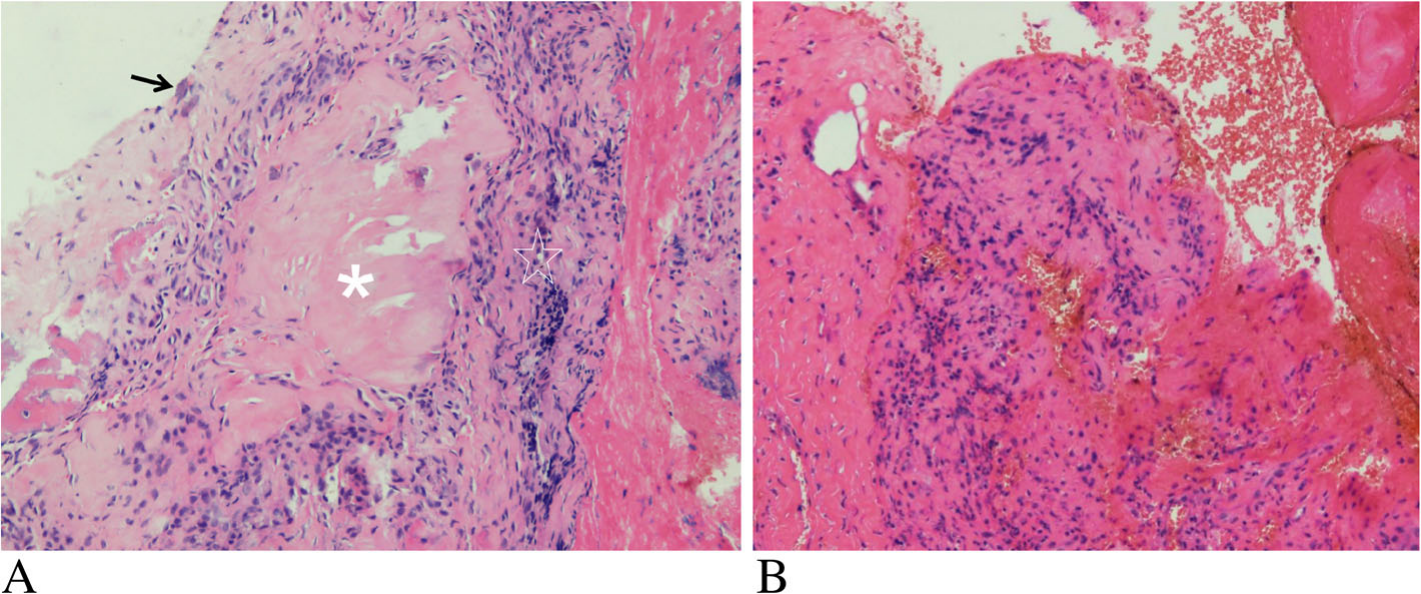
Histological section of a surgically resected specimen of the same patient. Hematoxylin and erosin (HE) staining (**a** and **b**) showing the massive deposition of uric acid crystals (**a**, asterisk) surrounded by granulomatous inflammation (**a**, pentagon), and few small calcium deposition (**a**, arrow), as well as fiber and angiogenesis with a large number of chronic inflammatory cells infiltration (**b**)

In this case, with clinical features, laboratory tests and imaging studies, physicians initially had difficulty in determining the lesions of C5–6; both infectious spondylodiscitis and spinal gout could not be ruled out. In terms of laboratory tests, it was difficult to judge that hyperuricemia was caused by peripheral gout or new lesions, and that the elevation of C-reactive protein was caused by gout or infection. As for the imaging studies of low specificity, radiologist was not completely sure of the etiology and such case was rarely met at that time; initially the imaging reported the first consideration was infectious spondylodiscitis. Under the circumstance of the lesions being not determined and nerve root symptoms, surgical treatment was performed, for the purpose of timely decompressing the spinal cord and providing conditions for the recovery of its function. Intraoperative findings showed that intervertebral disc of C5–6 was completely destructed and replaced by turbid fluid and the erosion of adjacent endplates and vertebrae. The pathological examination of the specimen demonstrated deposited uric acid crystals surrounded by granulomatous inflammation, as well as fiber and angiogenesis with a large number of chronic inflammatory cells infiltration (Fig. 4). Postoperatively, the lesions of C5–6 were definitely diagnosed as gout.

### Therapeutic intervention

Preoperatively, pharmaceutical treatment was given to relieve pain, nourish nerve and dehydrate [1]. To immediately decompress the nerve root symptoms, surgical treatment including anterior cervical corpectomy, debridement and fusion, and internal fixation of C5–6 was performed [1, 8, 11]. After surgery, the muscle strengths of limbs were significantly improved. Postoperatively, the new diagnosis was confirmed; then colchicine and prednisolone were respectively given 1 mg t.i.d and 10 mg t.i.d. whose dosage reduced when symptoms improved [1, 11]. Rehabilitation training including electronic biofeedback therapy, pneumatic compression, equipment training and electronic standing bed training was also performed [15].

### Follow-up and outcomes

About 3 months after surgery, the patient felt that pain of neck and shoulder was not obvious and there was no sensory abnormality in the upper limbs; the muscle strengths of limbs were all improved, with upper limbs grade 4 and lower limbs grade 2- to 4 in accordance with Medical Research Council (MRC) Scale for Muscle Strength [14]. The level of serum uric acid was well controlled between 336 umoles/l and 399 umoles/l (normal range, 208–428 umoles/l).

## Discussion and conclusions

In our case, gout-related cervical spine lesions were confirmed and a series of complete and continuous high-resolution images including multiplanar reconstruction (MPR), volume rendering (VR) CT images, plain and contrast-enhanced MR images together with exhaustive description were fully displayed. To the best of our knowledge, there have only been 10 such cases with images reported in the English-language literature searched in PubMed (Table 1) [3–11]; most of them just show incomplete and indistinct images. Due to that there were also reports that spinal gout had similar image appearance to infection [4, 5, 16, 17], the main imaging differentiation factors between these conditions should be further discussed.

**Table 1:** Case Reports of Tophaceous Gout in the Cervical Spine

	Age, gender	Known gout	Symptoms	Imaging findings	Lab test results	Histology	Outcome	Co-morbidities/ History
1. Duprez TP	59, M	+	Progressive impairment of walking	MRI: involvement of C3–6	UA: 9.9 mg/dl	At surgery: gouty tophi in the posterior longitudinal ligament	Improvement after surgery	N. D.
2. Yen HL	68, M	+	Neck pain and numbness and weakness of all four limbs	MRI: narrowing of the thecal sac at the C3–6 levels	UA: 12.1 mg/dl	Biopsy: presence of monosodium urate crystals	Improvement after surgery	Diabetes mellitus
3. Cabot J	76, F	N.D.	Neck pain and upper limb weakness	MRI: arthritis of the right C4–5 facet joint and the C7-T1 left facet joint	UA: 12.2 mg/dl CRP: 147 mg/l	CT-guided aspiration: monosodium urate crystals	Improvement with colchicine therapy	Diabetes mellitus, diabetic nephropathy, hypertension
4. Dharmadhikari R	66, F	–	Weakness of all four limbs, urinary continence	MRI: involvement of C3–6 and cord compression	UA: 12.6 mg/dl	Biopsy: presence of gout crystals	Death caused by bronchopneumonia after surgery	Atrial fibrillation, hypertension, moderate cardiac dysfunction
5. Yamamoto M	58, F	+	Polyarticular disability	CT: involvement of C4–7	UA: 9.4 mg/dl CRP: 85.5 mg/l	–	Improvement with medication	Chronic arthritis
6. Wendling, D	54, M	+	Inflammatory neck pain and CBN	MRI: involvement of C5 and C6	UA: 11.5 mg/dl CRP: 5 mg/l	N.D.	Full recovery with colchicine therapy	Hypercholesterolemia
7. Wendling, D	72, M	+	Acute neck pain, knee arthritis	MRI: C5-C6 discitis	UA: 4.1 mg/dl CRP: 93 mg/l	N.D.	Full recovery with colchicine therapy	Hypertension
8. Nunes EA	59, M	N.D.	Occipital pain, cervicalgia	MRI: involvement of the odontoid process	UA: 8.6 mg/dl	Biopsy: inflammatory granuloma and monosodium urate crystals	Full recovery after surgery	Polyarthritis, hypertension
9. Ng W	66, M	N.D.	Neck pain, weakness of upper limbs	MRI: involvement of C4–6 and cord compression	UA: 7.6 mg/dl CRP: 7.7 mg/l	Biopsy: presence of urate crystals	Improvement after surgery and medication	Diabetes mellitus, hypertension, chronic renal insufficiency
10. Cheng CW	23, M	+	Back pain, weakness of the lower limbs	MRI: epidural collection from C4 to T10	UA: 14.6 mg/dl CRP: 122.5 mg/l	Biopsy: inflammatory granuloma, urate crystals	Full recovery after surgery and medication	Chronic renal disease. Transverse myelitis

UA: uric acid, CRP: C-reactive protein, N.D.: not described, CBN: cervicobrachial neuralgia

Gouty involvement in intervertebral disc may be rarer than facet joint, which mostly mimics pyogenic discitis [5, 11, 16, 18], but it showed the opposite outcome in the reports reviewed in our article. Considering these 10 reports and including our case, 7 patients’ lesions involved discs and only 2 involved facet joints. As for sites of cervical involvement, spinal lesions of 7 patients were seen in the middle cervical segment; 3 of them located in the middle and lower cervical segment, and only 1 patient’s lesion located in the upper cervical segment. The lesions can occur in any segment of cervical spine, but are commonly seen in the middle cervical spine C3–6.

The imaging findings of our patient were initially difficult to tell if it was gout or infection. However, looking back on the images, we found that radiological abnormalities for spinal gout may have some difference with common infectious spondylosis according to literature report. Plain films sometimes appear false negative especially in the early stages and subsequent bone changes are advantageous but nonspecific feature of spinal gout [4, 6, 7, 11]. CT can help visualize the bone and soft tissue changes caused by tophi which appear as low-density area [6, 7, 9, 10]. The typical appearance related to these changes is characterized by punched-out erosion and well-defined margins without surrounding infiltrative changes; structures of vertebrae and endplates can remain unaffected and wedging changes of vertebral bodies are hardly seen [6–10]. MRI appears to be sensitive but nonspecific for the diagnosis of spinal gout, but MRI has an obvious advantage in manifesting abundant information of soft tissue in or around the gouty lesion [19–21]. In the literature review, 5 cases involving the cervical intervertebral discs with MR images was mainly low signal intensity on T1 and T2 weighted images [4, 5, 7, 9]. In 2 cases with enhanced MR images, the involved discs were enhanced significantly [4, 9]. And MR characteristics of 4 patients with tophus were intermediate-to-low signal intensity on T1-weighted images, and on T2-weighted images, the signal intensity varied from low to high [4, 7, 9, 10], which mostly coincided with previous studies [1, 19, 20]. After gadolinium enhancement, the tophi shows homogeneous or heterogeneous enhancement, mainly depending on volumes and distribution of calcification [16]. In the previous studies, the MRI findings have ranged from well-defined punched-out endplate erosive change and narrowing of intervertebral space to severe destructive and proliferative discovertebral changes. And no significant edema was found in the trabecular bone and soft tissue adjacent to the lesions. However, spinal cord edema can sometimes be seen resulting from the cord compression by lesions [5, 7, 10, 11].

Imaging manifestations of spinal gout is not specific and can resemble that of degenerative changes and infectious spondylodiscitis [4, 5, 18, 22, 23]. Therefore, it is necessary to exclude other probable etiologies. In patients with Modic type 1 vertebral disc changes, reactive sclerosis accompanied by marrow edema usually appears as hypointense on T1-weighted images and hyperintense on T2-weighted and STIR images in acute conditions, and its lesions generally show well defined boundary [24]. For pyogenic spinal infection, disc changes appear as invariably reduced height, non-anatomic T2 hyperintense and enhanced significantly; irregularity, destruction and enhancement of endplates and adjacent vertebral bodies can be seen as well. Inhomogeneous paraspinal inflammatory swelling is common and small abscesses with a limited area can also be formed in some cases [25]. However, vertebral lesions in spinal tuberculosis are usually extensive, causing collapse and malformation in the late stage. Bone destruction, dead bone, narrowing of intervertebral space, angulate deformity and cold abscess are the hallmarks of the disease and can help differentiate it from other conditions, and paraspinal collection occurs more commonly than in pyogenic infection and may be bilateral and disproportionately larger than the degree of bone destruction [26, 27]. In the cases of spinal brucellosis, the principal features in the sclerotic period are vertebral body hyperplasia, osteophyte proliferation, bony spines resembling “bird beaks” (that sometimes forms bony bridges) and endplate sclerosis; paraspinal soft tissue shadows and predural granulomas are more common than usual, and paravertebral abscesses are less common [27]. The main radiological differentiation factors between these conditions is the characteristics of the osteolytic lesions and changes of paravertebral tissues.

In recent years, a newer method, dual-energy CT (DECT), has been more commonly applied in the differential diagnosis of gout [3, 28, 29]. The sensitivity of DECT in identifying monosodium urate crystals up to 78–100% makes it more competitive than other methods of imaging. The urate depositions can be clearly seen on DECT, which is able to directly measure the size and volume of depositions [1]. Therefore, DECT apparently has diagnostic potential for patients with an unclear diagnosis or atypical clinical manifestations [22].

Clinically, it is necessary for physicians to break the boundary of the reasoning mechanism that local lesions in the intervertebral discs and adjacent vertebral endplates are just degenerative changes, infectious disease or tuberculosis; henceforth, gout should be higher on the list of differentials especially for the patients not responding to conservative measures. In laboratory tests, hyperuricemia and the elevated level of C-reactive protein were also uncertain that they were caused by gout of limbs or the new lesions, and the new lesions were gout or infection. Blood culture should be supplemented to exclude the possibility of infection when the lesion was difficult to define. Definite diagnosis of gout was most commonly made during surgery. In the 10 clinical cases, surgery was performed in 6 cases. An additional 1 case was diagnosed via needle aspiration. In 3 cases, the patients were diagnosed on the basis of blood culture, other clinical data, a history of gout and a rapid response to colchicine. Similar to the majority, etiology of our case was completely confirmed during surgery.

Treatment for spinal gout commonly includes conservative medication and surgery. Owing to the fact that data in most literature suggests that spinal gout may contribute to paraplegia, early intervention is needed after a definite diagnosis. Decompression surgery followed by pharmacological treatment of lowering serum uric acid may be the first treatment for the majority of patients [1]. If necessary, individual prophylaxis and therapeutic strategies can be provided as well.

Cervical spinal gout involving the disc and adjacent vertebral endplates is uncommon and may misunderstand degenerative changes and infectious spondylodiscitis. When such this condition with atypical symptoms and low specific imaging studies is encountered clinically, physician and radiologist should take the gouty spondylitis into account with a combination with previous history and clinical manifestations.

## Data Availability

All data generated or analysed during this study are included in this article and other published articles in the reference.

## Abbreviations

CT: Computed tomography
DECT: Dual-energy computed tomography
DM: Diabetes mellitus
MPR: Multiplanar reconstruction
MRI: Magnetic resonance imaging
STIR: Short time inversion recovery
VR: volume rendering
